# Temporal trends in outcome and patient characteristics in dilated cardiomyopathy, data from the Swedish Heart Failure Registry 2003–2015

**DOI:** 10.1186/s12872-021-02124-0

**Published:** 2021-06-18

**Authors:** Helen Sjöland, Jonas Silverdal, Entela Bollano, Aldina Pivodic, Ulf Dahlström, Michael Fu

**Affiliations:** 1 Department of Molecular and Clinical Medicine, Institute of Medicine, Sahlgrenska Academy, Sahlgrenska University Hospital/Östra Hospital, University of Gothenburg, Smörslottsgatan 1, 416 85 Gothenburg, Sweden; 2 Statistiska Konsultgruppen, Gothenburg, Sweden; 3 Department of Clinical Neuroscience, Institute of Neuroscience and Physiology, Sahlgrenska Academy, University of Gothenburg, Gothenburg, Sweden; 4 Department of Cardiology and Department of Health, Medicine and Caring Sciences, Linköping University, Linköping, Sweden

**Keywords:** Dilated cardiomyopathy, Secular trends, Prognosis, Phenotype, Treatment, Real life data

## Abstract

**Background:**

Temporal trends in clinical composition and outcome in dilated cardiomyopathy (DCM) are largely unknown, despite considerable advances in heart failure management. We set out to study clinical characteristics and prognosis over time in DCM in Sweden during 2003–2015.

**Methods:**

DCM patients (n = 7873) from the Swedish Heart Failure Registry were divided into three calendar periods of inclusion, 2003–2007 (Period 1, n = 2029), 2008–2011 (Period 2, n = 3363), 2012–2015 (Period 3, n = 2481). The primary outcome was the composite of all-cause death, transplantation and hospitalization during 1 year after inclusion into the registry.

**Results:**

Over the three calendar periods patients were older (*p* = 0.022), the proportion of females increased (mean 22.5%, 26.4%, 27.6%, *p* = 0.0001), left ventricular ejection fraction was higher (*p* = 0.0014), and symptoms by New York Heart Association less severe (*p* < 0.0001). Device (implantable cardioverter defibrillator and/or cardiac resynchronization) therapy increased by 30% over time (mean 11.6%, 12.3%, 15.1%, *p* < 0.0001). The event rates for mortality, and hospitalization were consistently decreasing over calendar periods (*p* < 0.0001 for all), whereas transplantation rate was stable. More advanced physical symptoms correlated with an increased risk of a composite outcome over time (*p* = 0.0043).

**Conclusions:**

From 2003 until 2015, we observed declining mortality and hospitalizations in DCM, paralleled by a continuous change in both demographic profile and therapy in the DCM population in Sweden, towards a less affected phenotype.

**Supplementary Information:**

The online version contains supplementary material available at 10.1186/s12872-021-02124-0.

## Introduction

Dilated cardiomyopathy (DCM) constitutes a subset of heart failure (HF) conditions, characterized by the presence of left ventricular dilatation and contractile dysfunction, which is not explained by abnormal loading conditions (hypertension or valvular heart disease) or coronary artery disease. DCM has an estimated contemporary prevalence of > 1 case per 250 individuals [1], accompanied by a serious prognosis, and a global increase of 27% during the last 10 years [2]. It makes up the most common indication for heart transplantation [3]. During recent decades, remarkable advances have been made in the treatment opportunities and diagnostic possibilities in HF. However, the phenotypic and prognostic evolvement of DCM over time is not fully known. Our group has shown increasing HF hospitalization in young adults in Sweden, with cardiomyopathy accounting for a substantial share [4]. Here, we present a report of patients with DCM from an unselected nationwide population of patients included in the Swedish Heart Failure Registry (SwedeHF) from 2003 to 2015 and describe their composition, and unfolding changes over time, relative to the calendar period for inclusion in the registry.

## Methods

### The Swedish Heart Failure Registry

SwedeHF is an internet-based registry, launched 2003, recording clinical details for all HF patients from participating centres, and described in detail previously [5]. The registry covers 65 of 75 hospitals, including approximately 50% of all HF patients treated in hospital settings. A clinically assessed diagnosis of HF, by the attending cardiologists, constitute the inclusion criterion for registration. The protocol, registration forms, and annual reports are available at http://www.swedehf.se. Approximately 80 variables were recorded, at hospital discharge or at an outpatient visit, to a web‐based database managed by the Uppsala Clinical Research Centre (http://www.ucr.uu.se/en).

### Patient selection and data

From January 1, 2003 until December 31, 2015, SwedeHF registered n = 69 537 patients with HF. Patients included in this study had received a clinical diagnosis of DCM either as an entry into the registry (diagnosis of DCM or primary etiology as DCM), or a diagnosis of DCM in the National Patient Register (see below). For this study the following exclusions were made: Patients who did not receive a clinical diagnosis of DCM (n = 61 578), death during index hospitalization (n = 67), heart transplantation performed before registration (n = 19), leaving a final sample of n = 7873 patients with DCM (11.3% of the total registry).

Baseline data included clinical characteristics, medical history, laboratory findings, pharmacologic, or device treatment [defined as implantable cardioverter defibrillator (ICD), cardiac-resynchronization therapy (CRT), or a combination thereof]. Socioeconomic data and comparative population statistics (for age and sex) were obtained from the Statistics Sweden database (https://www.scb.se/en), and co‐morbidities and mortality from the Swedish National Patient Register and the Cause‐Specific Death Register (http://www.socialstyrelsen.se). Diagnoses and/or cause of death were coded according to the International Classification of Diseases system *International Statistical Classification of Diseases and Related Health Problems, 10th Revision*. The SwedeHF registry and this study are in accordance with the 1964 Declaration of Helsinki and its amendments, and approved by a Swedish multisite ethics committee and the Central Ethical Review Board in Linköping, Sweden, respectively. Individual patient consent was not required for entry into the national register, but patients were informed of the procedure and allowed to opt out.

### Outcome measures

For comparative analyses over the course of time, the patients were divided into three calendar periods, from January 1st to December 31st of the respective year, with the objective of attaining a balanced distribution of patients included per calendar period: Period 1; 2003–2007, Period 2; 2008–2011, Period 3; 2012–2015. The outcome measures of this study were: mortality, heart transplantation, cardiovascular (CV) hospitalization, HF hospitalization, or all-cause hospitalization one year after registration in SwedeHF. A composite endpoint was constructed, as the primary endpoint, of all these 1-year outcomes together.

### Statistical analysis

For baseline characteristics, continuous variables are presented as mean ± standard deviation or median and inter‐quartile range (IQR), where appropriate, while categorical variables are presented as frequencies and percentages. Comparing the differences in characteristics among calendar periods, we used the Mantel–Haenszel *χ*^2^ test for dichotomous and ordered categorical variables, *χ*^2^ test for non‐ordered categorical variables, and the Jonckheere–Terpstra test for continuous variables. Crude event rates for the outcome measures were calculated as the number of events per 100 person‐years, using exact Poisson‐based 95% confidence intervals (CIs). Event rates adjusted for age and sex for the three studied calendar periods (2003–2007, 2008–2011, 2012–2015) were obtained using Poisson regression. Likewise, the adjusted yearly trends of incidences over time 2003–2015 were performed using Poisson regression retrieving relative risks (RR) and 95% CI from the analyses. The prognostic impact of baseline variables was analysed using Cox regression including interaction between the studied variable and the calendar period. First, adjustment was made for age and sex and in the final model additionally including New York Heart Association (NYHA) functional classification, left ventricular ejection fraction (LVEF), any device and hypertension. Missing values of NYHA (17.6%), LVEF (5.8%) and device (n = 1.4%) variables were handled as *unknown* category in the adjustments. For all tests, statistical significance was set to *p* ˂ 0.05 (two tailed). Analyses were performed, and artworks were created using SAS software, Version 9.4 (SAS Institute Inc., Cary, NC, USA).

## Results

### Clinical phenotype of DCM over time

The baseline characteristics over three calendar periods are presented in Table 1. Patients were older (mean 63.9, 64.9, 64.9 years, *p* = 0.022), and the proportion of females increased (mean 22.5%, 26.4%, 27.6%, *p* = 0.0001) over calendar periods. For clinical variables, the distribution of LVEF reached significantly higher values (*p* = 0.0014), and functional classification by NYHA showed less functional limitation (*p* < 0.0001) over time. Estimated glomerular filtration rates improved (*p* < 0.0001). The prevalence of most comorbidities (diabetes, atrial fibrillation, lung disease, etc.) were stable over time. However, hypertension (38.6%, 48.8%, 52.5%, *p* < 0.0001) and sleep apnea (2.9%, 4.2%, 6.2%, *p* < 0.0001) increased over time.

**Table 1:** Patient characteristics by calendar period 1–3 for all individuals with DCM

Variable	Total (n = 7873)	Period 1 2003–2007 (n = 2029)	Period 2 2008–2011 (n = 3363)	Period 3 2012–2015 (n = 2481)	*p* value
*Patient characteristics*					
Age (years)	64.6 (13.5)	63.9 (13.5)	64.9 (13.5)	64.9 (13.6)	0.022
*Sex*					
Female	2030 (25.8%)	456 (22.5%)	889 (26.4%)	685 (27.6%)	0.0001
*Smoking*					
Never	2548 (38.9%)	622 (37.8%)	1094 (39.1%)	832 (39.3%)	
Former	2730 (41.6%)	685 (41.6%)	1145 (41.0%)	900 (42.6%)	
Current	1279 (19.5%)	339 (20.6%)	557 (19.9%)	383 (18.1%)	0.095
*Clinical measurements*					
Weight (kg)	83.3 (19.6) n = 7385	82.9 (18.8) n = 1925	83.1 (19.7) n = 3117	84.0 (20.0) n = 2343	0.066
BMI (kg/m^2^)	27.4 (7.5) n = 4042	27.1 (5.2) n = 942	27.1 (5.6) n = 1490	27.8 (9.7) n = 1610	0.068
*BMI category (kg/m*^*2*^*)*					
≥ 30	1125 (27.8%)	238 (25.3%)	397 (26.6%)	490 (30.4%)	0.0029
Systolic blood pressure (mmHg)	121.1 (19.9) n = 7791	120.4 (20.4) n = 2005	121.4 (20.0) n = 3324	121.3 (19.2) n = 2462	0.066
Diastolic blood pressure (mmHg)	73.7 (12.3) n = 7778	73.3 (12.8) n = 2004	74.0 (12.3) n = 3314	73.4 (11.9) n = 2460	0.55
Heart rate (bpm)	73.9 (15.1) n = 7438	73.3 (14.9) n = 1711	73.9 (14.6) n = 3286	74.3 (15.8) n = 2441	0.16
Mean arterial pressure	89.5 (13.3) n = 7773	89.0 (13.7) n = 2001	89.8 (13.4) n = 3312	89.4 (12.9) n = 2460	0.22
*NYHA class*					
I	775 (12.0%)	198 (11.5%)	355 (12.4%)	222 (11.7%)	
II	3031 (46.7%)	712 (41.3%)	1335 (46.7%)	984 (51.7%)	
III	2427 (37.4%)	733 (42.5%)	1047 (36.6%)	647 (34.0%)	
IV	252 (3.9%)	80 (4.6%)	120 (4.2%)	52 (2.7%)	< 0.0001
*LVEF*					
< 30%	4455 (60.0%)	1192 (62.7%)	1917 (60.7%)	1346 (57.1%)	
30–39%	1700 (22.9%)	404 (21.2%)	707 (22.4%)	589 (25.0%)	
≥ 40%	1264 (17.0%)	306 (16.1%)	535 (16.9%)	423 (17.9%)	0.0014
*ECG*					
Sinus rhythm	4764 (61.4%)	1219 (61.2%)	2027 (61.2%)	1518 (61.9%)	
Atrial fibrillation/flutter	2117 (27.3%)	579 (29.1%)	931 (28.1%)	607 (24.8%)	
Pacemaker/other rhythm	872 (11.2%)	193 (9.7%)	353 (10.7%)	326 (13.3%)	0.0001
LBBB	1917 (29.6%)	467 (30.1%)	797 (28.9%)	653 (30.1%)	0.88
QRS width (ms)	121.1 (31.2) n = 5552	121.0 (32.0) n = 1441	119.1 (30.6) n = 2108	123.3 (31.0) n = 2003	0.0058
*Laboratory tests*					
Haemoglobin (g/L)	137.7 (16.9) n = 7801	137.9 (16.9) n = 2025	137.1 (16.8) n = 3359	138.3 (17.0) n = 2417	0.24
Potassium (mmol/L)	4.20 (0.42) n = 5254	4.20 (0.42) n = 641	4.19 (0.42) n = 2414	4.22 (0.42) n = 2199	0.054
Creatinine (µmol/L)	100.8 (49.3) n = 7836	105.1 (47.2) n = 2022	100.8 (50.1) n = 3359	97.3 (49.6) n = 2455	< 0.0001
NT-proBNP (ng/L)	4503 (6361) n = 3353	4365 (6651) n = 404	4675 (6385) n = 1352	4393 (6266) n = 1597	0.85
eGFR (ml/min/1.73m^2^)	71.0 (24.3) n = 7836	69.1 (24.7) n = 2022	71.0 (24.7) n = 3359	72.7 (23.1) n = 2455	< 0.0001
*Medical history from SwedeHF or patient register*					
Hypertension	3729 (47.4%)	784 (38.6%)	1642 (48.8%)	1303 (52.5%)	< 0.0001
Diabetes	1719 (21.8%)	426 (21.0%)	741 (22.0%)	552 (22.2%)	0.32
Atrial fibrillation	3458 (43.9%)	885 (43.6%)	1470 (43.7%)	1103 (44.5%)	0.56
Lung disease/COPD	1390 (17.7%)	349 (17.2%)	591 (17.6%)	450 (18.1%)	0.41
Stroke/TIA	814 (10.3%)	197 (9.7%)	336 (10.0%)	281 (11.3%)	0.068
Liver disease	290 (3.7%)	79 (3.9%)	128 (3.8%)	83 (3.3%)	0.32
Renal disease	316 (4.0%)	75 (3.7%)	141 (4.2%)	100 (4.0%)	0.60
Dialysis	38 (0.5%)	5 (0.2%)	19 (0.6%)	14 (0.6%)	0.14
Peripheral vascular disease	516 (6.6%)	124 (6.1%)	221 (6.6%)	171 (6.9%)	0.29
Sleep apnoea	360 (4.6%)	59 (2.9%)	140 (4.2%)	161 (6.5%)	< 0.0001
Malignant cancer within last 3 years	651 (8.3%)	164 (8.1%)	280 (8.3%)	207 (8.3%)	0.76
Musculoskeletal or connective tissue disorder	1008 (12.8%)	231 (11.4%)	448 (13.3%)	329 (13.3%)	0.073
*Medical treatment at discharge*					
ACEI	5716 (73.0%)	1503 (74.5%)	2495 (74.7%)	1718 (69.5%)	< 0.0001
ARB	1894 (24.5%)	456 (23.1%)	793 (24.0%)	645 (26.2%)	0.013
ACEI/ARB	7349 (93.7%)	1887 (93.4%)	3134 (93.6%)	2328 (94.1%)	0.34
Beta blockers	7179 (91.7%)	1806 (89.7%)	3068 (91.7%)	2305 (93.4%)	< 0.0001
MRA	3297 (42.2%)	890 (44.2%)	1296 (38.8%)	1111 (45.2%)	0.32
Digoxin	1367 (17.5%)	484 (24.1%)	593 (17.8%)	290 (11.7%)	< 0.0001
Statins	2968 (37.9%)	713 (35.4%)	1295 (38.8%)	960 (38.9%)	0.025
Loop diuretics	5697 (74.8%)	1515 (79.8%)	2456 (75.5%)	1726 (70.1%)	< 0.0001
Anticoagulants	3322 (42.5%)	868 (43.2%)	1391 (41.6%)	1063 (43.0%)	0.96
ASA	2835 (36.4%)	765 (38.1%)	1309 (39.3%)	761 (30.9%)	< 0.0001
Nitrates	620 (7.9%)	220 (11.0%)	260 (7.8%)	140 (5.7%)	< 0.0001
*Device treatment*					
Any device	1009 (13.0%)	234 (11.6%)	408 (12.3%)	367 (15.1%)	< 0.0001
None	6755 (87.0%)	1782 (88.4%)	2911 (87.7%)	2062 (84.9%)	
ICD without CRT	372 (4.8%)	75 (3.7%)	153 (4.6%)	144 (5.9%)	
CRT without ICD	269 (3.5%)	95 (4.7%)	115 (3.5%)	59 (2.4%)	
CRT with ICD	368 (4.7%)	63 (3.1%)	140 (4.2%)	165 (6.8%)	< 0.0001

For categorical variables n (%) is presented. For continuous variables Mean (SD)/n = is presented

*DCM* dilated cardiomyopathy, *BMI* body mass index, *ECG* electrocardiography, *NYHA* New York Heart Association, *LVEF* left ventricular ejection fraction, *LBBB* left bundle branch block, *NT-proBNP* N-terminal proB natriuretic peptide, *eGFR* estimated glomerular filtration rate, *SwedeHF* Swedish Heart Failure Registry, *COPD* chronic obstructive pulmonary disease, *TIA* transitory ischemic attack, *ACEI* angiotensin converting enzyme inhibitor, *ARB* angiotensin receptor blockade, *MRA* mineralocorticoid receptor antagonist, *ASA* acetylsalicylic acid, *ICD* implantable cardioverter-defibrillator, *CRT* cardiac resynchronisation therapy

### Treatment of DCM over time

As for HF treatment, there were notable changes over time: The use of device treatment increased (11.6%, 12.3%, 15.1%, *p* < 0.0001) as did treatment with beta-blockers and statins (*p* < 0.0001 and *p* = 0.025, respectively). Use of digoxin (24.1%, 17.8%, 11.7%), loop diuretics (79.8%, 75.5%, 70.1%), acetyl salicylic acid (ASA), and nitrates diminished (*p* < 0.0001 for all). Treatment with renin angiotensin system (RAS)-blockade was stable over time, although angiotensin receptor blockers (ARB) increased (*p* = 0.013), and angiotensin converting enzyme inhibitors (ACEI) decreased (*p* < 0.0001).

### Prognosis of DCM over time

Table 2 shows incident events during 1-year follow-up for each endpoint, first cumulative 1 year event rate for the whole time period, followed by calendar periods. Overall, event rates decreased over calendar periods for all outcomes except transplantation. The composite endpoint occurred in approximately half of the patients over 1 year (48.4% during the whole follow-up). The most prevalent outcome was hospitalization for any cause, in 40.8–47.0% of patients, depending on calendar period. The rate of transplantation was 0.7% for the whole timespan.

**Table 2:** Events during 1 year follow-up for all-cause mortality, composite endpoint and hospitalizations

Endpoint	Calendar period	n (%) events	Event rate per 100 person years (95% CI)	Event rate per 100 person years (95% CI) adjusted for age and sex
All-cause mortality	2003–2015	881 (11.2%)	12.0 (11.2–12.8)	8.9 (8.1–9.7)
	Period 1	254 (12.5%)	13.6 (12.0–15.4)	10.7 (9.3–12.2)
	Period 2	365 (10.9%)	11.6 (10.4–12.8)	8.3 (7.4–9.3)
	Period 3	262 (10.6%)	11.3 (10.0–12.8)	8.1 (7.1–9.3)
Transplantation	2003–2015	55 (0.7%)	0.8 (0.6–1.0)	0.4 (0.3–0.6)
	Period 1	10 (0.5%)	0.5 (0.3–1.0)	0.3 (0.1–0.5)
	Period 2	30 (0.9%)	1.0 (0.6–1.4)	0.5 (0.3–0.8)
	Period 3	15 (0.6%)	0.6 (0.4–1.1)	0.4 (0.2–0.6)
HF hospitalization	2003–2015	2406 (30.6%)	40.8 (39.2–42.5)	40.8 (39.2–42.4)
	Period 1	659 (32.5%)	44.8 (41.5–48.4)	45.0 (41.7–48.6)
	Period 2	1050 (31.2%)	41.7 (39.2–44.3)	41.6 (39.2–44.2)
	Period 3	697 (28.1%)	36.5 (33.9–39.3)	36.4 (33.8–39.2)
CV hospitalization	2003–2015	3091 (39.3%)	56.7 (54.7–58.8)	56.5 (54.5–58.5)
	Period 1	831 (41.0%)	61.4 (57.3–65.7)	61.8 (57.7–66.2)
	Period 2	1373 (40.8%)	59.7 (56.5–62.9)	59.2 (56.1–62.4)
	Period 3	887 (35.8%)	49.4 (46.2–52.8)	49.1 (45.9–52.4)
Any hospitalization	2003–2015	3540 (45.0%)	68.3 (66.0–70.5)	68.0 (65.8–70.3)
	Period 1	947 (46.7%)	73.8 (69.2–78.7)	74.6 (70.0–79.5)
	Period 2	1580 (47.0%)	72.6 (69.0–76.2)	72.0 (68.5–75.6)
	Period 3	1013 (40.8%)	58.7 (55.1–62.4)	58.2 (54.7–61.9)
Composite endpoint	2003–2015	3810 (48.4%)	73.5 (71.2–75.8)	72.9 (70.6–75.3)
	Period 1	1024 (50.5%)	79.8 (75.0–84.9)	80.6 (75.8–85.7)
	Period 2	1681 (50.0%)	77.2 (73.5–81.0)	76.2 (72.6–80.0)
	Period 3	1105 (44.5%)	64.0 (60.3–67.9)	63.1 (59.5–66.9)

Number and percentage of events during 1-year follow-up, unadjusted and adjusted event rates, overall and by calendar period for index date, for all-cause mortality, composite endpoint, and hospitalizations. *CI* confidence interval, *HF* heart failure, *CV* cardiovascular, composite endpoint is 1-year mortality, heart transplantation, and all hospitalizations; Period 1 is 2003–3007, Period 2 is 2008–2011, and Period 3 is 2012–2015

**Fig. 1:**
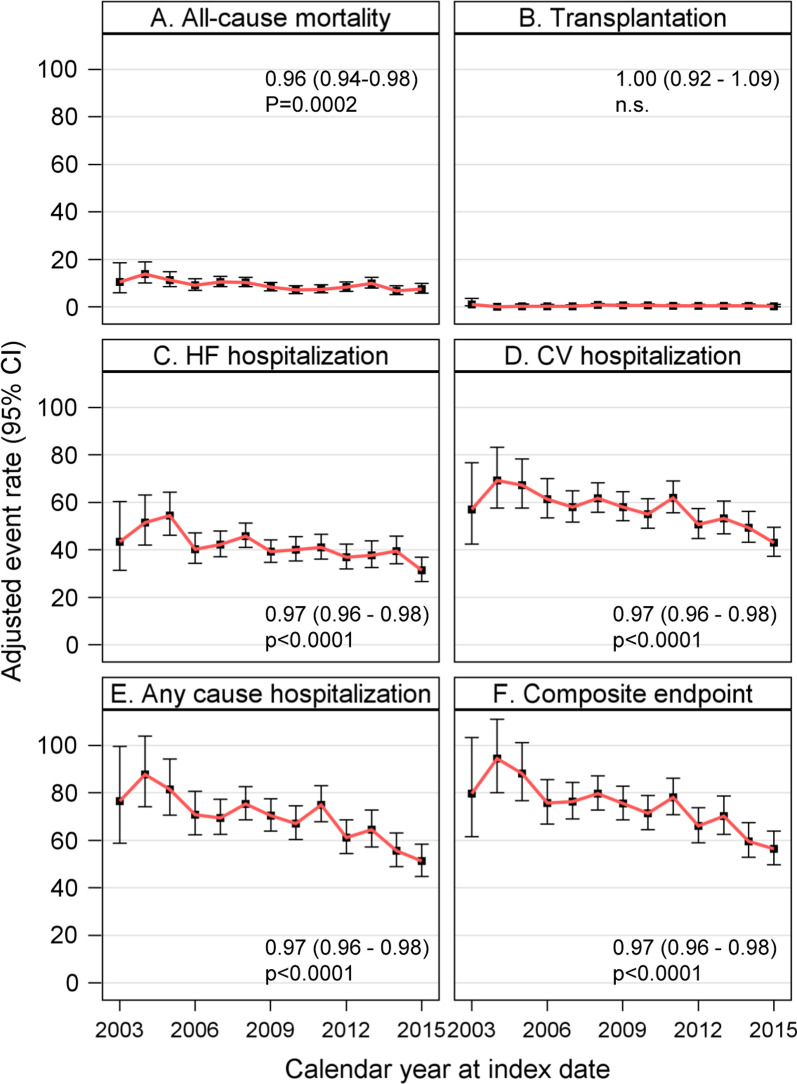
One year event rates for endpoints by index year (per 100 person years): Age-and sex-adjusted analysis of trend over time with relative risk (95% confidence interval) expressed by 1 year increase and *p* values. *CI* confidence interval, *HF* heart failure, *CV* cardiovascular

The 1 year age- and sex-adjusted event rates per 100 person years (95% CI) for all outcomes are presented (95% CI) in Fig. 1: all-cause mortality decreased over time, from 10.5 (6.0–18.6) year 2003 to 7.6 (5.8–9.8) during 2015, analysis for trend over time RR 0.96 [0.94–0.98 (95% CI)], *p* = 0.0002. Transplantation was stable over time (Fig. 1b), whereas HF hospitalization, CV hospitalization, hospitalization for any cause, and composite outcome decreased significantly RR 0.97 [0.96–0.98, (95% CI)], *p* < 0.0001 for all (Fig. 1c–f).

### Prognostic determinants for DCM over time

**Fig. 2:**
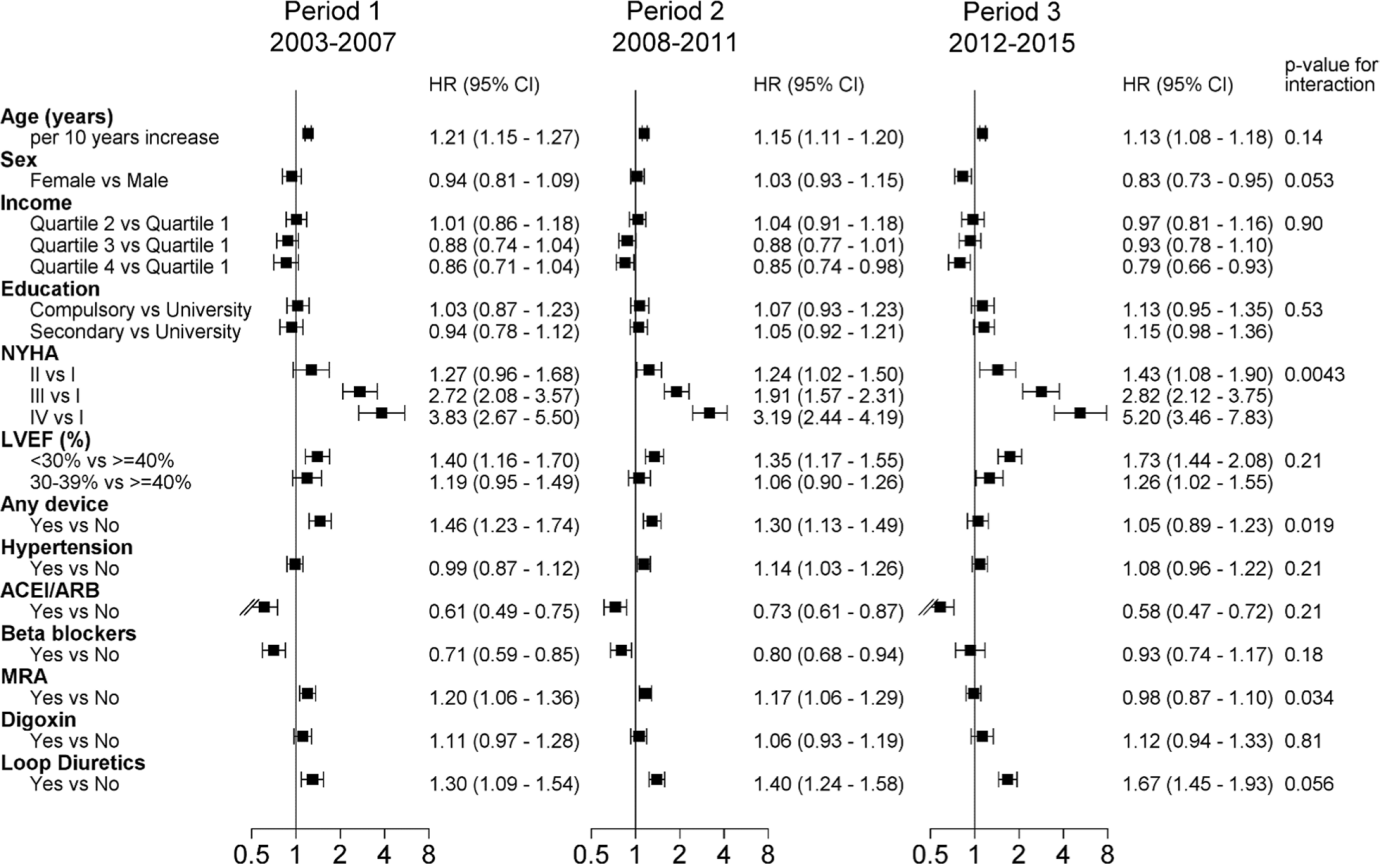
Risk of 1 year composite endpoint (death, heart transplantation, and any cause hospitalization) over calendar periods, and interaction with time, adjusted for age and sex. *HR* indicates hazard ratio, *NYHA* New York Heart Association functional class, *LVEF* left ventricular ejection fraction, *ACEI* angiotensin converting enzyme inhibitor, *ARB* angiotensin receptor blockade, *MRA* mineralocorticoid receptor antagonist

A set of variables were evaluated for association with outcome and interaction with time, adjusted for age and sex. Figure 2 shows a forest plot of hazard ratios (HR) for the composite endpoint for each calendar period, and interaction between the baseline variable and time (corresponding Additional file 1 for test of statistical independence with additional adjustments). Significant associations with worse outcome during all calendar periods were found for age, greater functional limitation by NYHA, lower LVEF, and treatment with loop diuretics. Correspondingly, a significant association with better outcome was observed for ACEI/ARB treatment. Age, NYHA class, and ACEI/ARB treatment remained independently associated with outcome after broader adjustment (Additional file 1).

Significant interaction with time occurred for NYHA class, device, and mineralocorticoid receptor antagonist (MRA) treatment (Fig. 2): the proportionally largest interaction with time was observed for NYHA class, as a worse functional class was associated with a marked increase in risk for a composite endpoint over time: for NYHA IV vs NYHA I, HR (95% CI): 3.83 (2.67–5.50) for Period 1, 3.19 (2.44–4.19) for Period 2, and 5.20 (3.46–7.83) for Period 3, *p* = 0.0043 for interaction. In contrast, for treatment with device, and for MRA, the risk for adverse outcome was increased during the earliest calendar period, decreased over time, and reached a similar risk as no device (*p* = 0.019), and no MRA use (*p* = 0.034) by the last calendar period: For device HR (95% CI): 1.46 (1.23–1.74) for Period 1, to 1.05 (0.89–1.23) for Period 3, and for MRA 1.20 (1.06–1.36) for Period 1, to 0.98 (0.87–1.10) for Period 3. Most clinical variables (age, sex, LVEF, hypertension), or treatments (ACEI/ARB, beta blockade, digoxin or loop diuretics) did not interact significantly with time. Additional adjustment for LVEF, NYHA, device and hypertension gave similar results, with somewhat attenuated interaction.

## Discussion

During the last decades we have seen important advances in the treatment of systolic HF and thus DCM. Here, we compare patients with DCM, from the SwedeHF registry included over three calendar periods. The strength of our study is the large unselected inclusion of all patients receiving a diagnosis of DCM from the participating units. However, our dataset constitutes an observational registry and no attempts to causal inference can be made. The patients included were identified based on the diagnosis given according to ICD-10-registration by the clinician. The LVEF reported in the SwedeHeart registry reflected the LVEF at the time of inclusion into the registry, which was not necessarily the LVEF at the time of diagnosis. We observed a less severe phenotype of DCM over time, and shifts in treatment, increases as well as decreases, paralleled changes in guideline recommendations. Mortality and hospitalizations decreased. However, the adversity of a low functional level was comparatively more pronounced over time.

### Clinical characteristics changed over time

In this study we observed that the DCM populations, included across three calendar periods, were less severely affected over time. They demonstrated better LVEF, less functional limitation, and better renal function at baseline, all being hallmarks of better myocardial function. We propose that greater availability to diagnostics and therapy over time, also led to inclusion of less severely affected patients. An older population, included over time, may partly be due to demographically longer life expectancy. However, the increase in female patients with DCM, exceeds the expected proportion relative to age and time period for the population (Statistics Sweden database), and was most likely explained by more female patients being affected. Then again, with more accessible and improved health care over time, a larger proportion of less affected patients may be included. Males are at higher risk of developing DCM and have a slightly worse prognosis in HF than women [2, 6]. Our results showed that men formed the majority of the affected over all three time periods, thus confirming the excess risk for men to develop DCM [2, 6]. Comparable studies of temporal trends in DCM are scarce, represented by cohorts from two tertiary Italian centers, in Trieste and Florence, also showing similar results across time, in support of a less affected phenotype, and a slightly increasing proportion of women (although n.s.) [7–9].

Most comorbidities were constant over time. However, the proportion of patients with hypertension increased considerably. Notably, hypertension was not assessed as a cause of DCM, but as a comorbidity. The reason is not clear, since diagnostic criteria for hypertension were unchanged over the duration of the study [10–12]. One explanation may be increased availability and awareness of blood pressure measurement. Most importantly, hypertension did not appear to affect DCM prognosis, which may also partly be due to more thorough and improved antihypertensive therapy per se. Finally, sleep apnea also increased. The enhanced risks of sleep apnea in HF are well established and hopes for new therapies with effects also on HF have abounded during recent decades. We may therefore speculate over increased attention to the diagnosis, rather than a true increase in prevalence. Unfortunately, the hopes for positive prognostic effects in HF of sleep apnea treatment have not been met [13, 14].

### Changes in therapy over time

Long being the cornerstone of HF therapy, treatment with digoxin and diuretics decreased considerably, in line with more restrictive recommendations in European HF guidelines [15–17]. The same was observed for ASA and nitrates, given a lesser role in chronic HF [15–17]. The high levels of RAS- and beta-blocker treatment agree with guidelines, at well over 90% each. Over time, treatment with ARB increased, corresponding to decreased treatment with ACEI, in line with changes in guidelines and establishment of ARB as a solid alternative to patients intolerant of ACEI [15, 17–19]. New evidence-based treatment for DCM during the study period was limited to device therapy [20–22], which increased most considerably, by 30% over time, although from a low level. Regrettably, the use of CRT for HF in Sweden is consistently lower than prognostic appraisals [23]. Importantly, indications for CRT have changed over time. On one hand, requirement of QRS duration has become more stringent from > 120 ms before, to presently > 130 ms. On another hand, CRT has received wider indications, from NYHA III–IV to II with time [15, 17, 24]. We propose that changes in treatment in DCM, during the period, were mostly explained by adherence to therapeutic guidelines. However, the use of device was disappointingly low also in DCM, and the unsuccessful implementation of guidelines regarding device treatment for HF in Sweden has been previously observed [23].

### Decrease in one year mortality and hospitalizations with time

One year mortality and hospitalizations decreased significantly over the calendar periods, whereas transplantation was stable. We suggest that less severe disease at onset and improved HF care may contribute to the improved outcome over time. This is supported by a lack of decrease in heart transplantations, which is a therapeutic choice, only indicated in the most severely ill. Heart transplantation was still required to the same extent over the whole duration of time. Recent data from the Trieste group also show improved survival over time [9] similar to our data.

### Risk factors associated with outcome changed over time

The first line of guideline directed medical therapy (GDMT) for HF (RAS-blockade, beta blockade, diuretics) did not interact with time for the composite endpoint. However, the interaction analysis showed that the relative role of device and MRA therapies in adverse outcomes diminished over time. Both constitute second line therapies, prescribed for non-responders when first line therapy does not sufficiently relieve symptoms [15, 17]. Interestingly, the increased risk for patients treated with device or MRA, diminished over time, and was neutral during the last calendar period. This gives the impression that early device or MRA treatment included only those at highest risk for adverse outcome, whereas our results correspond to extension of treatment to a broader range of patients over time. As for device therapy, the treatment increased, also indicating more widespread application to successively less severe cases. This is also supported by overall more severe HF towards earlier time periods (younger age, more severe NYHA functional class and lower LVEF). Also, treatment recommendations in therapeutic guidelines initially targeted the most severely affected patients: in the 2005 European HF guidelines MRAs were only mentioned as adjunct therapy in severe fluid overload, and device therapy was not included [16]. In the 2008 guidelines both MRAs and device (primarily CRT) therapy were recommended in severe HF (LVEF ≤ 35% and NYHA functional class III or IV) [15], but recommendations were not expanded to less symptomatic patients (NYHA functional class II) until 2012 [17], which is consistent with our results.

A slight but favorable outcome for women for the composite endpoint was seen only during the 3rd time period. Interpretation must be made with caution but does not contradict earlier studies [2, 6]. Better socioeconomic status, such as higher income and educational level, carry protective effects against HF [25]. However, our observations were quite discreet. For income a modest protective effect was seen during the later time periods for the highest income quartile, versus the lowest. We did not find any association between educational level and outcome, which confirms prior observations, once HF is present [26, 27]. It appears reasonable that socioeconomic advantages may reduce the risk of falling ill with serious disease but have a lesser impact when the disorder is already established. We must stress that we only had 1 year data follow-up, and endpoints were few, which represents limited power in the analysis. The differences in risk between groups for sex, income, and education were limited, and not consistent over time.

The majority of variables associated with outcome were well established CV risk factors, as expected. However, a couple of observations merit mentioning: the single pharmacologic treatment independently associated with decreased risk, during all calendar periods, was RAS-blockade. Even though causality cannot be decided, it underscores the choice of RAS-blockade as the preferred first treatment in DCM. Correspondingly, only loop diuretics were associated with an elevated risk during all calendar periods. Here, we speculate on a reverse causality: The most severe cases of DCM, requiring diuretic therapy, are also prone to worse outcome, which is compatible with previous findings in HF [28–30]. Likewise, the most severely symptomatic patients, in functional class NYHA IV, carried a comparatively higher risk with time. Clearly, the most severely symptomatic, clinically overlapping those in need of loop diuretics, also carry the highest risk of unfavorable outcome over time. This finding is also supported by consistently increased risk in the more extensive adjustment analyses (Additional file 1). Overall, the data support that the improvement in prognosis is partially due to less severe DCM phenotype with time.

A low NYHA functional level in DCM represents a serious prognostic sign [31, 32], and in our data, more expressly over time. Despite overall better prognosis, the enhanced relative risk associated with more pronounced HF symptoms and need of diuretic treatment, should be seen as a sentinel for worse prognosis in advanced DCM, today more than historically.

## Limitations

The SwedeHF database comprises an unselected large real-life cohort of clinically diagnosed DCM, from a broad array of community out-patient-clinics, community hospitals and university clinics. However, compared to tertiary referral centers for DCM the quality of registry data has limitations. The diagnosis was made by the attending physicians and reported to SwedeHF, whereas extended diagnostic procedures such as right heart catheterization or coronary angiograms, are not included in the registry. In this report, 1-year follow-up is reported, and long-time prognosis cannot be inferred. Moreover, possible selection bias may have been present at the time of initiation of the registry, around 2003, as only a limited number of patients were reported from a few tertiary referral university hospitals. During later years the SwedeHF registry includes approximately 50% of all HF in-patients in Sweden. As with all registry studies causality cannot be concluded. Missing data and misclassifications cannot be controlled, and analyses depend on the data provided. During the timespan of the study, economic and cultural changes in society at large may have confounding effects in comparative studies over time. For example, the ethnicity and origin of the patients were not available, and an increased immigration to Sweden over time may affect the composition of the population of patients and thus outcome.

## Conclusion

In this nationwide study of patients with DCM in Sweden, included during three calendar periods, from 2003 to 2015, we observed declining mortality and hospitalizations in parallel with a continuous change in the demographic profile in the DCM population in Sweden, towards a less effected phenotype.

## Supplementary Information

**Additional file 1.** Risk of 1 year composite endpoint (death, heart transplantation, and any cause hospitalization) over calendar periods, and interaction with time, adjusted for age, sex, functional classification by NYHA, LVEF, any device treatment, and hypertension. *HR* indicates hazard ratio, *NYHA* New York Heart Association functional class, *LVEF* left ventricular ejection fraction, *ACEI* angiotensin converting enzyme inhibitor, *ARB* angiotensin receptor blockade, *MRA* mineralocorticoid receptor antagonist.

## Data Availability

The datasets used and analysed during the current study (deidentified participant data) are available on reasonable request from the corresponding author.

## Abbreviations

DCM: Dilated cardiomyopathy
NYHA: New York Heart Association
HF: Heart failure
SwedeHF: Swedish Heart Failure Registry
ICD: Implantable cardioverter defibrillator
CRT: Cardiac-resynchronization therapy
CV: Cardiovascular
IQR: Inter-quartile range
CI: Confidence interval
RR: Relative risk
LVEF: Left ventricular ejection fraction
ASA: Acetyl salicylic acid
RAS: Renin angiotensin system
ARB: Angiotensin receptor blockers
ACEI: Angiotensin converting enzyme inhibitors
MRA: Mineralocorticoid receptor antagonist
GDMT: Guideline directed medical therapy
