# Supplementary figures and images for: Temporal trends in outcome and patient characteristics in dilated cardiomyopathy, data from the Swedish Heart Failure Registry 2003–2015
Source: BMC Cardiovasc Disord. 2021 Jun 18;21:307. doi: 10.1186/s12872-021-02124-0 (PMC8212489; doi:10.1186/s12872-021-02124-0)

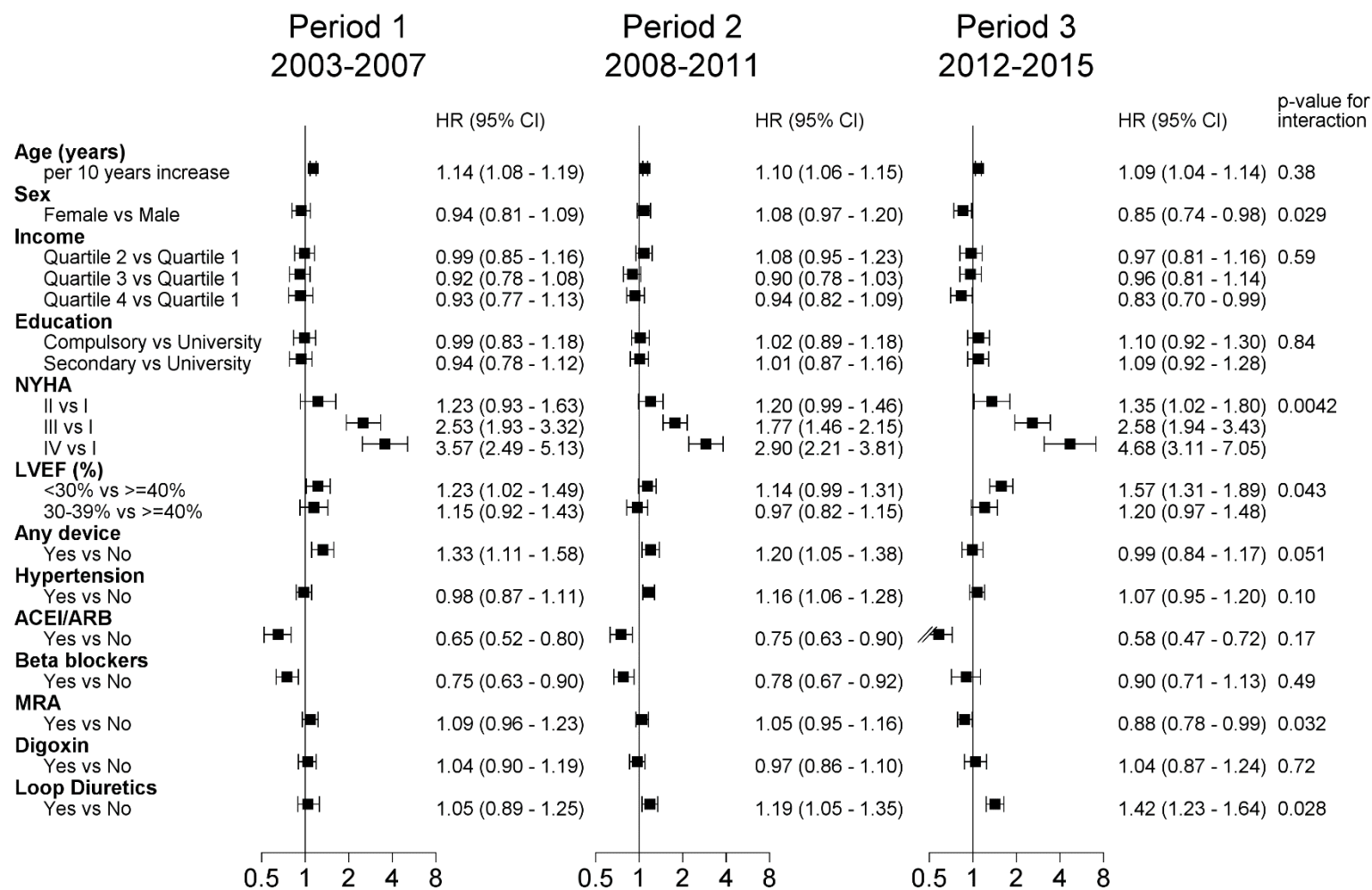

Supplement: Supplementary file 1 — Additional file 1. Risk of 1 year composite endpoint (death, heart transplantation, and any cause hospitalization) over calendar periods, and interaction with time, adjusted for age, sex, functional classification by NYHA, LVEF, any device treatment, and hypertension. HR indicates hazard ratio, NYHA New York Heart Association functional class, LVEF left ventricular ejection fraction, ACEI angiotensin converting enzyme inhibitor, ARB angiotensin receptor blockade, MRA mineralocorticoid receptor antagonist. [file 12872_2021_2124_MOESM1_ESM.pdf]
